# Differences in Provider Beliefs and Delivery of the 5As for Cigarette and Non-Cigarette Tobacco Use Between Two Types of Healthcare Centers Serving Rural and/or Medically Underserved Areas of Texas, US

**DOI:** 10.3390/healthcare13030338

**Published:** 2025-02-06

**Authors:** Ammar D. Siddiqi, Brian J. Carter, Maggie Britton, Tzuan A. Chen, Isabel Martinez Leal, Asfand B. Moosa, Teresa Williams, Kathleen Casey, Hector Sanchez, Lorraine R. Reitzel

**Affiliations:** 1 Department of Behavioral Science, University of Texas MD Anderson Cancer Center, 1155 Pressler St., Houston, TX 77030, USA; 2 Department of Management, Policy, and Community Health, School of Public Health, University of Texas Health Science Center at Houston, 1200 Pressler St., Houston, TX 77030, USA; 3 Feinberg School of Medicine, Northwestern University, 420 E Superior St., Chicago, IL 60611, USA; 4 Department of Psychological, Health, and Learning Sciences, University of Houston, 491 Farish Hall, Houston, TX 77204, USA; 5 HEALTH Research Institute, University of Houston, 4349 Martin Luther King Blvd., Houston, TX 77204, USA; 6 Department of Biology and Biochemistry, University of Houston, 4302 University Drive, Houston, TX 77004, USA; 7 Integral Care, 1430 Collier St., Austin, TX 78704, USA

**Keywords:** rural, tobacco intervention, medically underserved, 5As, cigarettes, non-cigarette tobacco, substance use treatment center, medical healthcare centers, tobacco use disparities, cancer prevention

## Abstract

Background/Objectives: Rural populations in the US bear a disproportionate burden of cancer mortality, which may be partly due to their elevated tobacco use and the limited receipt of tobacco use interventions in rural healthcare settings. Here, we examine providers’ use of the 5As (Ask, Advise, Assess, Assist, and Arrange), a brief tobacco cessation intervention, with their patients to assess intervention gaps. Methods: Provider practices in substance use treatment centers (SUTCs) and medical healthcare centers (MHCs), each serving rural and/or medically underserved areas (MUAs) of Texas, were compared. In total, 347 providers from 10 SUTCs (*n* = 174) and 9 MHCs (*n* = 173) responded to an anonymized survey about their cigarette and non-cigarette screening and intervention delivery, along with their perceived importance and workforce’s preparedness to help patients stop using tobacco. Linear mixed and generalized linear mixed models were used to assess differences between practices at SUTCs and MHCs. Results: More MHC than SUTC providers reported that cigarette and non-cigarette tobacco use cessation intervention were (respectively) important parts of their job (*p* = 0.0009; *p* = 0.0023) and that their workforce was prepared to help their patients quit tobacco (*p* = 0.0275), although less than half of all respondents endorsed preparedness. Relative to those at SUTCs, MHC providers reported higher rates of asking (SUTCs = 59.57% and MHCs = 77.21%; *p* = 0.0182) and advising (SUTCs = 45.34% and MHCs = 72.35%; *p* = 0.0017) their patients to quit cigarette smoking and advising them to quit non-cigarette tobacco products (SUTCs = 43.94% and MHCs = 71.76%; *p* = 0.0016). Conclusions: Overall, providers in both settings may benefit from greater preparation to deliver tobacco cessation care; needs were more prevalent within SUTCs than MHCs. Our findings can inform strategic planning to improve centers’ capacity to comprehensively address their patients’ tobacco use in rural/MUAs of Texas, US.

## 1. Introduction

Tobacco use contributes to nearly 20% of all cancer cases [1] and causes 480,000 deaths annually in the US, making it the nation’s leading cause of preventable death, disability, and disease [2,3]. Despite successful efforts by numerous public and private entities to reduce the use of conventional cigarettes [3,4], the burgeoning use of non-cigarette tobacco products (NCTPs, e.g., e-cigarettes and smokeless tobacco) [5,6] has caused the national prevalence of tobacco use among adult Americans to remain alarmingly high at 18.7% [4]. Although much of the long-term effects of some NCTPs are yet to be known [7], their markedly lower health risks compared to cigarettes have contributed to their elevated use [8,9], particularly among smokers unwilling to quit who may transition to NCTPs as a harm reduction strategy under guidance from providers and public health organizations [10,11]. NCTP’s proliferation is especially pronounced in rural areas of the US, where, for example, smokeless tobacco is used at a rate three times greater than in urban areas [12], and 26.2% of residents report using a tobacco product [4]. Such widespread usage affects not only the health of tobacco users but also some rural residents who do not use tobacco products through, for example, a greater risk of secondhand smoke exposure than their urban counterparts, particularly in their homes and workplaces [13]. Consequently, rural populations bear a disproportionate burden of morbidity and mortality from tobacco-related diseases [14,15].

Tobacco use in rural areas remains elevated for several reasons. First, access to medical care is often more limited in rural settings due to fewer hospitals, primary care providers, and specialists in these areas [16]. In conjunction with higher uninsured and poverty rates [17,18], these systemic barriers to healthcare make it less likely that rural residents will have contact with a provider from whom they can receive tobacco cessation care. Not surprisingly, rural residents are approximately three times less likely to receive any tobacco cessation treatment than urban residents [19]. This disparity helps contextualize why rural tobacco users are 50% less likely to have made an attempt to quit in the past 12 months [6,20] and have significantly lower odds of successfully quitting than their urban counterparts [21,22], thereby exacerbating tobacco-related health inequities.

Clinical practice guidelines recommend the use of the 5As (Asking patients about their tobacco use, Advising them to quit, Assessing their willingness to quit, Assisting them in quitting, and Arranging for follow-up), an evidence-based model for intervening with patients’ cigarette and/or NCTP use at every clinical encounter [23]. Delivery of all the 5As has been found to increase patients’ likelihood of using counseling, medication, or a combination of both [24], which can facilitate tobacco cessation [25]. Moreover, assisting patients in quitting and arranging for follow-up have been associated with improvements in quitting odds by 40% and 46% for smoking [26], respectively, with the delivery of the 5As also facilitating NCTP use cessation [27,28,29], though more work is needed in this area given the rapidly developing market of NCTP products. Despite this, a few dated studies conducted in the US demonstrate that the consistent delivery of the 5As is rare in rural settings [30,31]. Given the low receipt of tobacco cessation training by providers in rural areas, which has been reported as low as 11% in one study [32,33], the inconsistent delivery of the 5As may reflect low perceived importance of tobacco cessation care or low confidence in delivering such care, both of which have been found to subvert the delivery of the 5As [34]. The Health Beliefs Model and Social Cognitive Theory support this conjecture by establishing the influence that these cognitive factors can have on behavior (e.g., delivery of the 5As) [35,36].

Tobacco use disparities and intervention provision pertaining to rural populations are of particular concern in Texas, USA. While there is no universally accepted definition of rural in the US [37], various federal- and state-level definitions have been developed to address program- and regulation-specific needs. Some definitions are made at the county level, whereas others do not follow any municipal boundaries. Furthermore, rural is not always explicitly defined; instead, it may refer to any place that is not urban. Finally, definitions change over time. For example, in 2010, the US Census Bureau defined urban areas as having populations of 50,000 or more and urban clusters as areas with populations of 2500 to 24,999 [38]. In 2020, an area qualified as urban if it had “at least 2000 housing units or… a population of at least 5000” [39]. In both cases, rural was defined as “all population, housing, and territory not included within an urban area” [38,39]. The latest definition by the Office of Management and Budget (OMB) designates counties as Metropolitan (i.e., with a core urban area of 50,000 or more people), Micropolitan (i.e., with a core urban area of 10,000–49,999 people), or neither [40]. The Health Resources and Services Administration (HRSA) leverages definitions from the US Census Bureau and OMB adds its own parameters and creates a definition of rural areas for the purposes of determining eligibility for rural health grants [41,42]. Owed largely to its geographical size and population, Texas is the US state with the largest rural population (4.7 million rural residents) according to the 2020 census [43]. Additionally, maps and lists published by the Texas State Office of Rural Health and the Texas Department of Housing and Community Affairs classify between 177 and 194 (~70–76%) of Texas’ 254 counties as rural [44,45].

Most people living in rural areas also reside in areas with limited access to healthcare, making poor health outcomes inevitable; by one estimate, a staggering 80% of rural America is medically underserved [46]. HRSA defines medically underserved in three ways. First, it identifies areas with shortages of providers (called Health Professional Shortage Areas) in three disciplines: primary medical care, dental care, and mental health care. Second, it identifies medically underserved areas as geographic areas (e.g., counties, urban census tracts, etc.) with a shortage of primary medical care providers. Third, it identifies medically underserved populations as geographic areas with a shortage of primary medical care services for specific populations (e.g., individuals experiencing homelessness, with low income, who are Medicaid eligible, etc.) [47,48]. For the purposes of this paper, these areas are referred to collectively as Medically Underserved Areas (MUAs). Texas has 74 counties that are without a hospital, and over 100,000 Texans live in a county without a single primary care physician [49], contributing to its designation as the state with the second most MUAs [50]. The widespread inaccessibility of healthcare in these areas, along with documented inconsistent tobacco use screening practices in Texas healthcare organizations [51,52], diminish the likelihood that patients will receive tobacco cessation care. Nonetheless, little is known about the actual delivery of the 5As to patients using cigarettes or NCTPs in rural counties and/or MUAs of Texas.

Although prior studies have assessed tobacco screening, use of the 5As, and provider beliefs on tobacco cessation in rural medical settings, they are not generalizable to rural Texas because they were conducted in other geographical regions or urban areas of the US and, in some cases, other countries with different healthcare systems (e.g., in Australia and Canada, which have public/universal healthcare systems) [30,31,32,33]. Furthermore, these works did not differentiate provider practices in addressing patients’ cigarette and NCTP use, which is important based on the elevated use of NCTPs in rural areas [12,53]. Additionally, little is known about how care provision may differ in different types of healthcare centers, such as between medical healthcare centers (MHCs) and substance use treatment centers (SUTCs) (collectively referred to herein as “healthcare centers”). This is of interest because limited access to healthcare in rural areas heightens the importance of ensuring tobacco use screening and intervention delivery happens at every healthcare center, regardless of its scope of care. Moreover, this information can inform workplace programs to improve tobacco use screening and care delivery to patients, including by identifying settings most in need of intervention and by guiding the tailoring of intervention components to match needs. Ultimately, this information can be leveraged to address the tobacco-related health inequities experienced in rural and/or MUAs.

The current study redresses gaps in the literature by examining tobacco use assessment (screening) delivery to patients and the use of the 5As for conventional cigarette smoking and NCTPs, respectively, in MHCs and SUTCs that serve rural counties and/or MUAs of Texas. Additionally, it assesses providers’ beliefs about the importance of and their workforce’s preparedness to provide tobacco cessation care. Understanding provider tobacco intervention practices and beliefs in this setting will help to further contextualize tobacco use disparities by ascertaining the status of tobacco dependence care within rural counties and/or MUAs in Texas.

## 2. Materials and Methods

### 2.1. Procedures and Participants

Project staff compiled contact lists of healthcare centers (MHCs and SUTCs) in Texas, US, from internet searches, professional organization listservs, past work, etc. Recruitment was carried out largely through direct email, accompanied by a 1-page flyer detailing a comprehensive tobacco-free workplace program (TFWP) called Taking Texas Tobacco Free [54], which has focused on various recruitment targets (e.g., types of healthcare centers, urban versus rural areas, etc.) since its inception in 2013. Eligibility criteria for participation in the TFWP described herein included being an MHC or a SUTC in the state of Texas that self-identified as being located in and/or serving individuals from rural counties or MUAs in Texas and a willingness to participate in the TFWP to build or improve their tobacco use screening and cessation intervention capacity. As such, the present iteration of Taking Texas Tobacco Free was aptly titled Taking “Rural” Texas Tobacco Free. Additional recruitment strategies included team attendance as a vendor at professional organization meetings/conferences for healthcare center stakeholders (e.g., Texas Association of Rural Health Clinics [55], Texas Association of Community Health Centers [56], Texas Association of Addiction Professionals [57], etc.), where the TFWP was promoted via an exhibitor table with representatives available to answer questions and, in some cases, a 1-page flyer placed in attendee bags and/or oral presentation of the program to attendees. Interested individuals exchanged contact information with representatives and/or completed an online Qualtrics survey where they responded to an item asking if their healthcare center was located in, and/or served individuals from, rural or MUAs of Texas.

This study, which bridged most of the project team’s university affiliation change, was approved by the Internal Review Board of the University of Houston and later the Quality Improvement Assessment Board at the University of Texas MD Anderson Cancer Center. Data were collected from August 2021 through March 2024 via an electronic Qualtrics form from the healthcare centers that agreed to participate in the TFWP. Overall, 19 healthcare centers (9 MHCs and 10 SUTCs) participated in the TFWP during the cited data collection period.

After acquiring information about the healthcare center’s characteristics from leadership and prior to the receipt of any TFWP services from the team, the “pre-implementation” (i.e., baseline) survey was administered to employees. A cover letter describing the purpose of the study and key elements of informed consent preceded the survey items. The center leadership or designated Program Champion (an employee/s assigned by center leadership to work with the Taking Rural Texas Tobacco Free staff) emailed the survey link to their employees. The survey requested information about the healthcare center’s affiliation and queried whether the respondent was a general employee with no direct patient care provision or a direct service provider, defined as “someone who typically has one or more of the following credentials (and other similar credentials): [nurse practitioner, licensed vocational nurse, registered nurse, advanced practice nurse, certified nursing assistant, medical assistant, qualified mental health professional, doctor of medicine, licensed chemical dependency counselor, licensed social worker], etc. Direct service providers might also have titles like recovery coach, patient navigator, or peer support specialist”. The survey cover letter also indicated that respondents could receive a USD 10 Amazon gift card for completing the survey if their employer allowed it, directing them to another survey where they could leave their contact information for receipt of the e-gift card. Aside from the employees’ selection of their healthcare center affiliation from a drop-down list and self-identification as a direct service provider or general employee, no other possible identifying information was collected in the survey.

Overall, 542 employees from 9 MHCs and 490 employees from 10 SUTCs responded to the pre-implementation survey. This represented an average response rate of 73.61% of employees within the participating MHCs and 74.63% of employees within the participating SUTCs, according to information provided by their leadership (see Section 2.2.1). The analytic sample for the current study was narrowed to the 173 direct service providers (hereafter, providers) from MHCs and the 174 providers from SUTCs who were among the responding employees. This is because only direct service providers were asked about their tobacco cessation screening and care practices, as they were the only employees who provided this care. The corresponding average response rate was 49.19% of providers within participating MHCs and 49.45% of providers within participating SUTCs.

### 2.2. Measures

#### 2.2.1. Healthcare Center Characteristics

Descriptive healthcare center characteristics provided by center leadership were as follows: (1) the number of counties served; (2) the number of unique patients seen annually; (3) the number of unique visits each year; (4) the number of full and part-time employees; (5) the number of direct service providers; (6) whether the healthcare center provided tobacco cessation services or resources (yes vs. no); (7) whether they referred patients to the Texas Tobacco Quitline (yes vs. no); (8) whether they offered health promotion material on tobacco cessation (yes vs. no); and (9) whether the healthcare center had a comprehensive tobacco-free workplace policy, defined as disallowing tobacco use indoors and on the center’s property (yes vs. no).

#### 2.2.2. Provider Perceptions About Tobacco Use Intervention

Two items assessed providers’ perceptions of the importance of tobacco use care provision: (1) “Smoking cessation counseling is an important part of my job” and (2) “Non-cigarette tobacco use cessation counseling is an important part of my job”. A binary variable was used in analyses whereby responses of strongly disagree, somewhat disagree, or neither agree nor disagree were compared with somewhat agree or strongly agree for each item. This approach is similar to that used in other studies [58,59].

Providers answered the following item: “In your opinion, how prepared are the direct service providers in your workplace to help their patients aged 16 years or older quit tobacco use?”. This item was rated on a 5-point Likert scale ranging from not at all prepared to do so to completely prepared to do so. For analytic purposes, endorsements of not at all prepared to do so, somewhat unprepared to do so, or neither prepared nor unprepared were compared with endorsements of somewhat prepared to do so or completely prepared to do so.

#### 2.2.3. Provider Tobacco Use Intervention Practices

*Last month patient contacts.* Providers were asked to self-report the following: (1) the number of patients aged 16 years or older who they saw last month and (2) the number of patients aged 16 years or older who they saw last month who were new to their caseload. The survey was designed such that answers to the latter could not exceed the answers to the former. Providers were also asked (3) the number of patients aged 16 years or older who they saw last month who were conventional cigarette smokers and (4) the number of patients aged 16 years or older who they saw last month who used other (non-cigarette) tobacco products (including but not limited to e-cigarettes or vaping products). The answers to (3) and (4), respectively, could not exceed the response provided for (1) by survey design. The provider’s answer to the most relevant of each of these items was automatically piped into the wording of the items assessing tobacco use assessment delivery to new patients, use of the 5As for cigarette smoking, and the use of the 5As for NCTP use. In the descriptions below, this is referred to as “[# piped in]”.

*Tobacco use assessment delivery to new patients*. Providers who reported seeing at least one new patient last month answered an item assessing their delivery of tobacco use assessments: “You said you saw [# piped in] new patients aged 16 years or older in the last month. How many of these patients were given a comprehensive Tobacco Use Assessment (includes information like patient’s smoking status, smoking history, cigarettes, or packs smoked per day, number of years smoked, number of years since quitting smoking, etc.)?” Responses to this item were used to calculate the percentage of new patients 16 years or older to whom providers gave tobacco use assessments using simple division.

*Use of the 5As for cigarette smoking*. Providers who saw at least one patient last month answered items about their use of the 5As: (i) “You said you saw [# piped in] patients aged 16 years or older in the last month. How many of these patients did you:” (1) “Ask whether they smoked cigarettes?”; providers who saw at least one patient who used conventional cigarettes responded to the following: (ii) “You said you saw [# piped in] patients aged 16 years or older who were conventional cigarette smokers in the last month. How many of these patients did you:” (2) “Advise to quit smoking?”; (3) “Assess interest in making a smoking quit attempt?”; (4) “Assist to make a quit attempt (e.g., referral to the Texas Tobacco Quitline, on-site or offsite referrals, provided a direct intervention like counseling, medication, nicotine replacement therapy)?”; (5) “Arrange a follow-up contact to discuss progress with quitting?”. Responses to these items were used to calculate the percentage of patients that providers asked about cigarette use and the percentage of smokers to whom providers delivered the remaining 5As using simple division.

*Use of the 5As for NCTP use*. Providers who saw at least one patient last month answered items regarding their use of the 5As: (i) “You said you saw [# piped in] patients aged 16 years or older in the last month. How many of these patients did you:” (1) “Ask whether they used other non-cigarette tobacco products (including but not limited to e-cigarettes and vapes)?”; providers who saw at least one patient who used NCTPs responded to the following: (ii) “You said you saw [# piped in] patients aged 16 years or older who were users of other non-cigarette tobacco products in the last month (including but not limited to e-cigarettes and vapes). How many of these patients did you:” (2) “Advise to quit?”; (3) “Assess interest in making a quit attempt?”; (4) “Assist to make a quit attempt (e.g., referral to the Texas Tobacco Quitline, on-site or offsite referrals, provided a direct intervention like counseling, medication, nicotine replacement therapy)?”; (5) “Arrange a follow-up contact to discuss progress with quitting?”. Responses to these items were used to calculate the percentage of patients that providers asked about NCTP use and the percentage of NCTP users to whom providers delivered the remaining 5As using simple division.

### 2.3. Data Analyses

Data on healthcare center characteristics were reported using descriptive statistics. Comparisons between center types (i.e., MHCs vs. SUTCs) on these variables were assessed using Mann–Whitney tests for continuous variables and chi-square tests for binary variables. Mann–Whitney tests were used due to the small number of centers included in this study. To account for the nested data structure of providers within healthcare centers, linear mixed models and generalized linear mixed models (binomial distribution, logit link, and variance components for the variance matrix) were conducted with completed data for continuous and binary variables, respectively. Statistical significance was designated at *p* < 0.05. All analyses were conducted using SAS version 9.4.

## 3. Results

### 3.1. Assumptions

Diagnostic tests and checks for parametric assumptions for linear mixed models and generalized linear mixed models (i.e., linearity, normality, and homoscedasticity) were within acceptable limits.

### 3.2. Healthcare Center Characteristics

Together, the 19 participating healthcare centers served patients from 123 of Texas’ 254 counties (48.43%). Overall, they, on average, treated 6095 unique patients yearly via 21,471 annual visits and had 86 employees (62 of whom, on average, were direct service providers). MHCs served marginally significantly more patients yearly than SUTCs (*Z* = −2.0772; *p* = 0.053). See Table 1.

About 74% of centers provided tobacco cessation services or resources to patients, 42% referred patients to the Texas Tobacco Quitline, and 58% offered health promotion material on tobacco cessation. About half (47%) of the centers had a comprehensive tobacco-free workplace policy. The results from chi-square tests showed that there were no statistically significant differences between MHCs and SUTCs in terms of service/resource provision or policy variables.

### 3.3. Provider Perceptions About Tobacco Use Intervention

Of 347 direct service providers (173 from MHCs and 174 from SUTCs), 53.60% agreed that smoking cessation counseling is an important part of their job, with a significantly greater percentage of MHC providers (65.90%) agreeing than SUTC providers (41.38%; *p* = 0.0009). Similarly, 51.59% of all providers agreed that NCTP use cessation counseling is an important part of their job, with a significantly greater percentage of MHC providers (63.01%) agreeing than SUTC providers (40.23%; *p* = 0.0023). Overall, 48.41% of respondents agreed that the direct service providers at their healthcare center are at least somewhat prepared to help their patients quit tobacco use, with a significantly greater percentage of MHC providers (56.07%) agreeing than SUTC providers (40.80%; *p* = 0.0275). See Table 2.

### 3.4. Providers’ Tobacco Use Intervention Practices

Of the 347 providers surveyed, 90% (*n* = 313) reported seeing at least one patient aged 16 years or older in the prior month (MHCs: *n* = 155 (89.59%); SUTCs: *n* = 158 (90.80%)).

#### 3.4.1. Tobacco Use Assessment Delivery to New Patients

Of the 313 providers who reported seeing at least one patient last month, 92% (*n* = 288) reported that at least one of them was a new patient for them (MHCs: *n* = 149; SUTCs: *n* = 139). These providers reported giving 49.23% of their new patients a comprehensive tobacco use assessment, with a significantly greater percentage of new MHC patients (M(SD) = 62.72% (45.84%)) receiving a tobacco use assessment than SUTC patients (M(SD) = 34.77% (45.90%); γ = 29.64 (95% CI: 15.13, 44.15); *p* < 0.0001).

#### 3.4.2. Use of the 5As for Cigarette Smoking

Providers who saw at least one patient aged 16 years or older last month (*n* = 313), including both new and existing patients, reported asking 68.31% of them if they smoked cigarettes, with a significantly greater percentage of MHC patients being asked than SUTC patients (77.21% vs. 59.57%; γ = 14.36 (95% CI: 2.46, 26.26); *p* = 0.0182).

About 79% of providers (*n* = 247) had seen at least one patient in the last month who used cigarettes. These providers reported advising 58.68% of them to quit, assessing 51.90% of them about their interest in quitting, assisting 35.87% of them in making a quit attempt, and arranging a follow-up for 22.79% of them. A significantly greater percentage of MHC patients (72.35%) were advised to quit than SUTC patients (45.34%; γ = 26.38 (95% CI: 10.05, 42.71); *p* = 0.0017). See Table 3.

#### 3.4.3. Use of the 5As for NCTP Use

Providers who saw at least one patient aged 16 years or older last month (*n* = 313), including both new and existing patients, reported asking 62.48% of them whether they used NCTPs, with no significant differences between MHCs and SUTCs.

About 80% of providers (*n* = 251) had seen at least one patient in the last month who used NCTPs. These providers reported advising 58.02% of them to quit, assessing 52.84% of them about their interest in quitting, assisting 33.58% of them in making a quit attempt, and arranging a follow-up for 22.53% of them. A significantly greater percentage of MHC patients (71.76%) were advised to quit than SUTC patients (43.94%; γ = 27.08 (95% CI: 10.39, 43.78); *p* = 0.0016). See Table 4.

## 4. Discussion

The current work examined healthcare providers’ beliefs about tobacco cessation care, their screening practices, and their use of the 5As for tobacco use, a brief evidence-based intervention that is recommended by best practice guidelines to use with patients at every healthcare visit [23]. This is the first study, to our knowledge, to focus on healthcare centers providing services in rural counties or MUAs in Texas and to compare responses between two types of centers where rural or medically underserved patients obtain care. Further, the work adds to the extant literature by examining provider intervention provisions for cigarette smoking and NCTP use separately. The findings indicated that, relative to SUTC providers, MHC providers were significantly more likely to endorse greater prioritization and workforce preparedness in providing tobacco cessation care. Additionally, MHC providers reported using the 5As with a greater proportion of their patients relative to SUTC providers; in some cases, these differences were statistically significant (i.e., in asking about cigarette smoking and advising cigarette smokers and NCTP users, respectively, to quit). Overall, this work builds on existing literature documenting the use of the 5As for cigarette smoking in other rural areas of the US and reveals disparities in intervention delivery and provider beliefs between healthcare center types in Texas.

Although the results suggest that MHC providers deliver tobacco cessation care more consistently than SUTC providers, they also illuminate ample room for improvement in tobacco use care in both settings. For example, at least a third of providers in MHCs and almost two-thirds of SUTC providers did not believe that smoking and NCTP use cessation was an important part of their job. Though causation cannot be assessed in the present study, this may have been actualized in only 56% of MHC providers and 41% of SUTC providers reporting that their workforce was prepared to help their patients quit tobacco. This seems shocking, particularly for SUTCs where a primary mission is to often treat multiple types of substance dependencies and wherein providers should be trained to motivate and manage patients’ attempts to quit them. However, overall building tobacco care provision in both settings is paramount to addressing tobacco use disparities, given that tobacco use is 50% greater in rural than urban areas [12].

Prior work demonstrates how implementing TFWPs within healthcare centers can improve intervention delivery for patients using cigarettes [60] and NCTPs [58], counseling and medication provision [61], and employees’ tobacco-related knowledge and beliefs about treating patients’ tobacco use [58,62]. However, persistent challenges to delivering tobacco cessation care include providers’ reports of unfamiliarity with community resources, limited capacity/time to provide this care, and lack of knowledge of how to address tobacco use [51], the latter of which was queried and confirmed in the present study. These obstacles may pose challenges to implementing tobacco cessation care and TFWPs in healthcare centers serving rural counties and/or MUAs, particularly due to the known barrier of limited access to health-related resources [63], workforce shortages, and rampant employee turnover [64,65] found within them. Nevertheless, the results suggest significant gaps in the use of even the briefest of public health interventions—the 5As—in healthcare centers for tobacco use, which is the leading cause of preventable death and disability in the US [2]. Moreover, the ubiquity of accessible cessation services, such as the Texas Tobacco Quitline, which offers free evidence-based therapies (nicotine replacement therapy and counseling) to residents in any geographical area of Texas, should enable provider intervention via quitline referral, which does not require extensive time [66]. However, in this sample, only 36% of cigarette smokers and 34% of NCTP users were offered assistance to quit by their providers. This may be due to poor provider knowledge on tobacco cessation. A prior study reported that more than a third of Texas-based providers endorsed that their center is unfamiliar with the Texas Tobacco Quitline and half did not know that they provide nicotine replacement therapy to callers [67]. Limited knowledge of the quitline, in addition to low self-efficacy and perceived importance of offering tobacco cessation counseling, may subvert tobacco cessation intervention delivery. This is supported by two Texas-based studies where providers with greater knowledge of referral options, higher self-efficacy in treating tobacco use, and a higher prioritization of tobacco use counseling were more likely to deliver the 5As to patients who used cigarettes and NCTPs [34,59]. In light of this, the gaps in the use of evidence-based tobacco cessation practices and limited provider preparedness to help their patients quit tobacco demonstrate a need for these healthcare centers to build capacity and increase knowledge to improve their provision of tobacco care to patients. This may be achieved by participation in a TFWP (if possible) with the use of a train-the-trainer program for resiliency to staff turnover [68] and through regular tobacco-related educational opportunities that are easily accessible, such as those offered by the American Lung Association and the Smoking Cessation Leadership Center, among others.

Addressing tobacco dependence has historically been overlooked in SUTCs despite the patients they serve being twice as likely to use tobacco products than the general US population [69]. This, in part, stems from the limited availability of cessation care services and the poor uptake of tobacco-free policies in this setting [70]. In the current study, a smaller percentage of patients in SUTCs received a tobacco use assessment or the 5As, and fewer providers endorsed the importance of tobacco cessation care delivery in comparison to those at MHCs. Overall, only about half of healthcare centers in the current study thoroughly screened new patients with a tobacco use assessment—a cornerstone of health history intake and the basis on which referral to lung cancer screenings should be made. Tobacco use assessment rates were especially low in SUTCs, where only about 35% of new young adult and adult patients received one. These disparities may be contextualized by misconceptions SUTC providers have about tobacco. Prior studies have found that SUTC providers believe that tobacco can be an effective coping mechanism [71] and is not a real drug of significant concern [72] and that cessation will jeopardize patient sobriety from other substances [73]. These widely held beliefs are contrary to empirical findings that concurrent tobacco use can exacerbate mental illnesses, increase stress [74,75], and jeopardize sobriety from non-nicotine substances [76,77,78]. Prior work also suggests barriers to tobacco care provision in rural SUTCs (relative to urban SUTCs) include having fewer providers with advanced educational backgrounds, limited specialized treatment offerings, and severe staffing challenges [79,80]. Having a smaller proportion of staff with advanced educational training, for example, has been associated with offering fewer evidence-based treatment techniques in SUTCs [81]. Moreover, poor funding and staffing in SUTCs limit their capacity, which, in turn, may hinder their ability to offer tobacco cessation care. As a result, SUTCs serving rural counties and/or MUAs should develop strategies for recruiting and retaining employees with greater educational experience, such as by providing them with improved benefits and investing in capacity building and employee training (e.g., the use of online curricula and tele-mentoring) [64,82]. Some of these challenges and strategies to overcome them may be applicable to rural MHCs as well, inasmuch as they may be attributable to rural setting commonalities [83,84], but more research is needed.

The Health Beliefs Model and Social Cognitive Theory posit that human behavior is shaped, in part, by beliefs about the importance of health-promoting behavior and self-efficacy to perform the behavior, respectively [35,36]. Consequently, low perceived importance and low confidence in providing tobacco cessation care among providers can subvert the delivery of the 5As [34]. With significantly more providers at MHCs endorsing the importance and workforce preparedness in providing tobacco cessation care to their patients, it was expected that MHC providers would more consistently provide interventions for cigarette and NCTP use. Indeed, self-reported intervention delivery was consistent with these predictions, as evidenced by significantly greater delivery of ask and advise for cigarette use and advise for NCTP use in MHCs compared to SUTCs. These findings reinforce that TFWP interventions should be designed to address these factors to ultimately enhance providers’ tobacco cessation care delivery [60,68,85], tailored to the setting in which they are implemented. For example, SUTCs may benefit from more targeted education that explains how to deliver concurrent treatment for tobacco dependence and other substance use disorders and the evidence base behind and benefits of such approaches, given the pervasive misconceptions that exist about tobacco dependence [76,77,78]. Providers in rural areas and/or MUAs may require more comprehensive knowledge about free evidence-based resources to effectively support their patients (e.g., Texas Tobacco Quitline), who are more likely to be uninsured [18] and experience poverty [17] than patients being seen by providers in urban areas.

Prior work, though not focused on rural areas, suggests that patients may be less likely to receive brief interventions for NCTP use compared to cigarette use [58,60], potentially due to harm reduction approaches [10,11]. In the current study, the most marked difference in any of the 5As in this regard was for asking about use (62.48% of patients were asked for NCTP use versus 68.31% about cigarette use); however, these differences were slight and not assessed for statistical significance. Additionally, assessing interest in quitting NCTP use was greater than for cigarette use (52.84% of patients assessed versus 51.90%), but again, these appear to be minimal differences. It may be that the use of the 5As in our sample for both NCTP and cigarette use was more aligned than has been reported previously due to the recent emergence of a host of new NCTPs that are of particular interest to providers [86], a greater understanding that harm reduction approaches carry risk [10,11], or it may reflect the disproportionate use of NCTPs in rural populations [12,53] that makes NCTPs more relevant to the providers participating in this study. In any case, interventions to bolster provider provision of tobacco screening and cessation care would necessarily include robust education on the health risks associated with NCTPs and clinical practice guidelines, ensuring they can effectively counsel patients on the dangers of these products and provide them with evidence-based support to quit all forms of tobacco. Prior work supports that such education has improved providers’ tobacco-related beliefs, self-efficacy, perceived barriers, and NCTP use intervention delivery [58] and may be effective in further addressing misconceptions that prevent providers from addressing the disproportionate use of NCTPs in this setting [12].

### Study Limitations, Strengths, and Future Directions

Limitations of this study include the use of a convenience sample of healthcare centers that agreed to participate (at a later date than survey collection) in a TFWP [54]. Providers at participating centers may have been more inclined to respond to solicitation because they worked in a center interested in enhancing their tobacco cessation services, potentially leading to a selection bias in the study results. Additionally, providers at participating centers may differ from providers at centers not interested in TFWP implementation in terms of their beliefs and practices surrounding tobacco use. In that case, the results presented herein may reflect more favorable outcomes (e.g., greater perceived importance and delivery of care) to the extent that participation in a TFWP reflects a greater interest and commitment to tobacco use care. On the other hand, centers not interested in TFWP implementation may already be well-equipped to handle tobacco use care delivery.

While Texas has a sizeable number of rural counties and MUAs that enable meaningful results from this work, there may be limited generalizability of findings to other regions, where healthcare practices and patient demographics may differ. Future work should seek to include a broader range of healthcare centers from other states to provide a more representative understanding of tobacco cessation beliefs and care practices in rural and MUAs. Relatedly, a limitation of this work is that healthcare center contacts were asked whether they were located in and/or serving rural and/or MUAs. This reliance on self-reporting may affect the accuracy of the data regarding their service areas. However, we would expect that, considering funding opportunities tied to these designations (e.g., for rural health grants from HRSA) [42], healthcare centers know and should be able to accurately relay this information. Here, centers reported to us all counties they served; however, given that designations for rural and MUAs change depending on the source, some are not county-based, as well as the fact that designations change over time, tracking and verifying the true reach proves challenging. Future work should select a single definition at the inception of their project and track this information systematically throughout program implementation. However, a limitation to this approach is that implementation timelines can be lengthy, especially in resource-scarce areas, sometimes well exceeding a year. During this time, designations may change. Consequently, the expected implementation timeline should be considered when selecting a source.

Another limitation of this study is that providers’ beliefs were self-reported and may not be entirely representative of the true importance they place on cigarette and NCTP cessation counseling or their workforce’s preparedness to help their patients quit tobacco. Providers’ responses may have been influenced by social desirability, where they provided responses that they believed were expected or acceptable [87]. Furthermore, recall bias may have affected the accuracy of responses if providers had difficulty recalling past intervention delivery [88]. Although honest and accurate responding cannot be guaranteed, it was facilitated by designing surveys to be anonymous, notifying respondents in a survey cover letter that individual responses would not be shared with center leadership, and only asking about past-month intervention delivery. Additionally, there are other intervention practices for tobacco-using patients (e.g., motivational interviewing, 5Rs, etc.) that may be more appropriate than the 5As when working with patients who may be apprehensive about quitting tobacco [23]; these were not included in the present work. There is also a need to further investigate factors that may result in providers’ failure to deliver the 5As at every patient contact (e.g., favoring the use of harm reduction approaches, potentially legitimate rationales for not delivering the 5As at every contact such as by patient request or the need to focus on more salient issues like suicidality). Likewise, knowing more about the characteristics of the providers in each setting (e.g., years in practice, sex, and educational background) and their patients could help to further contextualize differences between healthcare center types in their provision of tobacco use care. A more comprehensive examination of responding providers and the full scope of their tobacco intervention practices (as well as their effectiveness) is needed. Finally, the current study was cross-sectional and not intended to examine causation. Future work in this area will use longitudinal designs to describe TFWP implementation and intervention outcomes.

## 5. Conclusions

Our findings suggest that, relative to their MHC counterparts, providers at SUTCs that serve rural counties and/or MUAs were less likely to see tobacco screening and intervention as important, report adequate preparation for these activities, and deliver some key aspects of evidence-based tobacco cessation care to their patients. However, greater compliance with best practice guidelines may be needed in both settings to mitigate tobacco use and disease disparities and can be promoted by training and encouraging employees to routinely provide tobacco cessation care to their patients. Educating providers about the dangers of both cigarettes and NCTPs is needed to ensure that they take a comprehensive approach to addressing tobacco dependence that is inclusive of all tobacco products. TFWP interventions that include provider education should be tailored to each setting; the differences identified between SUTCs and MHCs in this work suggest that there are more tobacco-related care deficits in the former setting that could inform implementation strategies used therein. More research is needed to determine the representativeness of findings from this Texas-based study under different parameters.

## Figures and Tables

**Table 1:** Select leadership-reported pre-implementation characteristics of medical healthcare centers (MHCs) and substance use treatment centers (SUTCs) serving rural and/or medically underserved areas in Texas, US, agreeing to participate in a comprehensive tobacco-free workplace program (***n*** = 19).

Select Center Characteristics	All Centers (*n* = 19)	MHC (*n* = 9)	SUTC (*n* = 10)	Statistic	*p*-Value
	Mean (SD) Range				
of counties served	7.89 (11.51) 1–40	2.56 (1.67) 1–6	12.70 (14.43) 1–40	−1.4143	0.1743
of unique patients/yearly †	6094.56 (10,688.54) 21–46,000	6102.89 (4255.18) 1000–12,139	6086.22 (14,988.79) 21–46,000	−2.0772	0.0532
of total patient visits/yearly †	21,470.89 (28,909.46) 21–98,000	20,127.78 (11,386.81) 1750–40,000	22,814.00 (40,524.87) 21–98,000	−1.5903	0.1302
of employees (inclusive of direct service providers)	85.68 (136.40) 3–600	68.00 (60.31) 15–205	101.60 (182.71) 3–600	0.6940	0.4965
of direct service providers	61.79 (83.66) 2–350	58.67 (52.75) 9–170	64.60 (107.27) 2–350	0.8169	0.4247

Note: Mann–Whitney tests were conducted for results presented in Table 1. † Missing information for 1 SUTC.

**Table 2:** Direct service providers’ perceptions of the importance of tobacco cessation care and the preparedness of their healthcare center’s workforce to deliver this care to patients at rural medical healthcare centers (MHCs) and substance use treatment centers (SUTCs) in Texas, US, prior to their healthcare center’s participation in a comprehensive tobacco-free workplace program (*n* = 347).

	All Providers (*n* = 347)	MHC Providers (*n* = 173)	SUTC Providers (*n* = 174)	γ (95% CI)	*p*-Value
	*n* [%]				
Smoking cessation counseling is an important part of my job. †					
Yes	186 [53.60]	114 [65.90]	72 [41.38]	0.22 (0.09, 0.36)	**0.0009**
No	161 [46.40]	59 [34.10]	102 [58.62]		
Non-cigarette tobacco use cessation counseling is an important part of my job. †					
Yes	179 [51.59]	109 [63.01]	70 [40.23]	0.20 (0.07, 0.33)	**0.0023**
No	168 [48.41]	64 [36.99]	104 [59.77]		
In your opinion, how prepared are the direct service providers in your workplace to help their patients aged 16 years or older quit tobacco use?					
Somewhat or completely prepared	168 [48.41]	97 [56.07]	71 [40.80]	0.14 (0.02, 0.26)	**0.0275**
<Somewhat or completely prepared *	179 [51.59]	76 [43.93]	103 [59.20]		

Note: Generalized linear mixed models were conducted for results in Table 2. † Yes = somewhat agree or strongly agree; No = strongly disagree, somewhat disagree, or neither agree nor disagree. * = not at all prepared to do so, somewhat unprepared to do so, or neither prepared nor unprepared. Differences between MHCs and SUTCs of *p* < 0.05 are **bolded**. Responses represent providers at 9 MHCs and 10 SUTCs.

**Table 3:** Direct service providers’ use of the 5As (Ask, Advise, Assess, Assist, and Arrange) with patients who smoked cigarettes over the last month at rural medical healthcare centers (MHCs) and substance use treatment centers (SUTCs) in Texas, US, prior to their healthcare center’s participation in a comprehensive tobacco-free workplace program (*n* = 313).

	All Providers (*n* = 313)	MHC Providers (*n* = 155)	SUTC Providers (*n* = 158)	γ (95% CI)	*p*-Value
	Mean (SD)				
% of patients asked about their smoking status	68.31 (42.91)	77.21 (38.43)	59.57 (45.35)	14.36 (2.46, 26.26)	**0.0182**
**Of patients you saw who smoked cigarettes, % that were…**	**All Providers** (*n* = 247) *	**MHC Providers** (*n* = 122) *	**SUTC Providers** (*n* = 125) *	**γ (95% CI)**	***p*** **-Value**
	**Mean (SD)**				
Advised to quit?	58.68 (46.48)	72.35 (40.69)	45.34 (48.04)	26.38 (10.05, 42.71)	**0.0017**
Assessed for interest in quitting?	51.90 (44.90)	57.67 (42.39)	46.26 (46.71)	6.64 (−9.62, 22.89)	0.4216
Assisted to quit?	35.87 (44.61)	41.94 (45.16)	29.95 (43.42)	7.44 (−8.93, 23.81)	0.3711
Arranged for a follow-up to discuss progress?	22.79 (39.36)	26.52 (40.64)	19.14 (37.86)	8.15 (−4.31, 20.60)	0.1987

Note: Linear mixed models were conducted for results presented in Table 3. Differences between MHCs and SUTCs of *p* < 0.05 are **bolded**. Responses represent providers at 9 MHCs and 10 SUTCs. * Sample size was reduced to only providers who saw at least one patient in the last month aged 16 years or older who smoked cigarettes.

**Table 4:** Direct service providers’ use of the 5As (Ask, Advise, Assess, Assist, and Arrange) with patients who used non-cigarette tobacco products (NCTPs) over the last month at rural medical healthcare centers (MHCs) and substance use treatment centers (SUTCs) in Texas, US, prior to their healthcare center’s participation in a comprehensive tobacco-free workplace program (*n* = 313).

	All Providers (*n* = 313)	MHC Providers (*n* = 155)	SUTC Providers (*n* = 158)	γ (95% CI)	*p*-Value
	Mean (SD)				
% of patients asked about their NCTP use status	62.48 (45.09)	69.49 (42.82)	55.59 (46.32)	11.21 (−1.23, 23.65)	0.0772
**Of patients you saw who used NCTPs, % that were…**	**All Providers** (*n* = 251) *	**MHC Providers** (*n* = 127) *	**SUTC Providers** (*n* = 124) *	**γ (95% CI)**	***p*** **-Value**
	**Mean (SD)**				
Advised to quit?	58.02 (47.72)	71.76 (42.59)	43.94 (48.74)	27.08 (10.39, 43.78)	**0.0016**
Assessed for interest in quitting?	52.84 (47.28)	57.63 (46.30)	47.93 (47.96)	7.85 (−9.24, 24.93)	0.3663
Assisted to quit?	33.58 (45.24)	38.25 (46.12)	28.80 (43.99)	6.77 (−10.83, 24.37)	0.4490
Arranged for a follow-up to discuss progress?	22.53 (39.58)	27.00 (41.87)	17.95 (36.70)	10.82 (−2.74, 24.38)	0.1172

Note: Linear mixed models were conducted for results presented in Table 4. Differences between MHCs and SUTCs of *p* < 0.05 are **bolded**. Responses represent providers at 9 MHCs and 10 SUTCs. * Sample size was reduced to only providers who saw at least one patient in the last month aged 16 years or older who used NCTPs.

## Data Availability

The data presented in this study are available upon request from the corresponding author. The data are not publicly available because the study is ongoing.

## References

[1] Islami F., Marlow E.C., Thomson B., McCullough M.L., Rumgay H., Gapstur S.M., Patel A.V., Soerjomataram I., Jemal A. (2024). Proportion and Number of Cancer Cases and Deaths Attributable to Potentially Modifiable Risk Factors in the United States, 2019. CA A Cancer J. Clin..

[2] CDC Current Cigarette Smoking Among Adults in the United States. https://www.cdc.gov/tobacco/php/data-statistics/adult-data-cigarettes/index.html.

[3] U.S. Department of Health and Human Services (2014). The Health Consequences of Smoking—50 Years of Progress: A Report of the Surgeon General.

[4] Cornelius M.E., Loretan C.G., Jamal A., Davis Lynn B.C., Mayer M., Alcantara I.C., Neff L. (2023). Tobacco Product Use Among Adults—United States, 2021. MMWR Morb. Mortal. Wkly. Rep..

[5] Mamudu H.M., Adzrago D., Dada O., Odame E.A., Ahuja M., Awasthi M., Weierbach F.M., Williams F., Stewart D.W., Paul T.K. (2023). Examining Disparities in Current E-Cigarette Use among U.S. Adults before and after the WHO Declaration of the COVID-19 Pandemic in March 2020. Int. J. Environ. Res. Public Health.

[6] Creamer M.R., Wang T.W., Babb S., Cullen K.A., Day H., Willis G., Jamal A., Neff L. (2019). Tobacco Product Use and Cessation Indicators Among Adults—United States, 2018. MMWR Morb. Mortal. Wkly. Rep..

[7] Yammine L., Tovar M., Yammine N.A., Becker C., Weaver M.F. (2024). E-Cigarettes and Youth: The Known, the Unknown, and Implications for Stakeholders. J. Addict. Med..

[8] Balfour D.J.K., Benowitz N.L., Colby S.M., Hatsukami D.K., Lando H.A., Leischow S.J., Lerman C., Mermelstein R.J., Niaura R., Perkins K.A. (2021). Balancing Consideration of the Risks and Benefits of E-Cigarettes. Am. J. Public Health.

[9] Colilla S.A. (2010). An Epidemiologic Review of Smokeless Tobacco Health Effects and Harm Reduction Potential. Regul. Toxicol. Pharmacol..

[10] FDA The Relative Risks of Tobacco Products. https://www.fda.gov/tobacco-products/health-effects-tobacco-use/relative-risks-tobacco-products#:~:text=as%20a%20whole.-,Are%20E%2DCigarettes%20a%20Lower%2DRisk%20Alternative%20to%20Cigarettes%3F,nicotine%2C%20which%20is%20highly%20addictive.

[11] Toll B.A., Smith T.T., King B.A. (2024). Nicotine E-Cigarettes: Considerations for Healthcare Providers. Nat. Med..

[12] Cornelius M.E., Loretan C.G., Wang T.W., Jamal A., Homa D.M. (2022). Tobacco Product Use Among Adults—United States, 2020. MMWR Morb. Mortal. Wkly. Rep..

[13] Vander Weg M.W., Cunningham C.L., Howren M.B., Cai X. (2011). Tobacco Use and Exposure in Rural Areas: Findings from the Behavioral Risk Factor Surveillance System. Addict. Behav..

[14] Henley S.J., Anderson R.N., Thomas C.C., Massetti G.M., Peaker B., Richardson L.C. (2017). Invasive Cancer Incidence, 2004–2013, and Deaths, 2006–2015, in Nonmetropolitan and Metropolitan Counties—United States. MMWR Surveill. Summ..

[15] Roberts M.E., Doogan N.J., Kurti A.N., Redner R., Gaalema D.E., Stanton C.A., White T.J., Higgins S.T. (2016). Rural Tobacco Use across the United States: How Rural and Urban Areas Differ, Broken down by Census Regions and Divisions. Health Place.

[16] National Rural Health Association About NRHA. https://www.ruralhealth.us/about-us/about-rural-health-care.

[17] U.S. Department of Agriculture Data Show U.S. Poverty Rates in 2019 Higher in Rural Areas than in Urban for Racial/Ethnic Groups..

[18] Cheeseman Day J. Health Insurance in Rural America. https://www.census.gov/library/stories/2019/04/health-insurance-rural-america.html.

[19] Ramsey A.T., Baker T.B., Pham G., Stoneking F., Smock N., Colditz G.A., James A.S., Liu J., Bierut L.J., Chen L.-S. (2020). Low Burden Strategies Are Needed to Reduce Smoking in Rural Healthcare Settings: A Lesson from Cancer Clinics. Int. J. Environ. Res. Public Health.

[20] Bittencourt L., Rubenstein D., Noonan D., McClernon F.J., Carroll D.M. (2024). Smoking Quit Attempts and Associated Factors Among Rural Adults Who Smoke Daily in the United States. Nicotine Tob. Res..

[21] Parker M.A., Weinberger A.H., Eggers E.M., Parker E.S., Villanti A.C. (2022). Trends in Rural and Urban Cigarette Smoking Quit Ratios in the US From 2010 to 2020. JAMA Netw. Open.

[22] Northridge M.E., Vallone D., Xiao H., Green M., Blackwood J.W., Kemper S.E., Duke J., Watson K.A., Burrus B., Treadwell H.M. (2008). The Importance of Location for Tobacco Cessation: Rural–Urban Disparities in Quit Success in Underserved West Virginia Counties. J. Rural Health.

[23] Fiore M., Jaén C., Baker T., Bailey W., Benowitz N., Curry S., Dorfman S., Froelicher E., Goldstein M., Healton C. Treating Tobacco Use and Dependence: 2008 Update. http://www.tobaccoprogram.org/clientuploads/documents/Consumer%20Materials/Clinicians%20Systems%20Mat/2008-Guidelines.pdf.

[24] Kruger J., O’Halloran A., Rosenthal A.C., Babb S.D., Fiore M.C. (2016). Receipt of Evidence-Based Brief Cessation Interventions by Health Professionals and Use of Cessation Assisted Treatments among Current Adult Cigarette-Only Smokers: National Adult Tobacco Survey, 2009–2010. BMC Public Health.

[25] U.S. Department of Health and Human Services Increase Use of Smoking Cessation Counseling and Medication in Adults Who Smoke—TU-13.

[26] Park E.R., Gareen I.F., Japuntich S., Lennes I., Hyland K., DeMello S., Sicks J.D., Rigotti N.A. (2015). Primary Care Provider-Delivered Smoking Cessation Interventions and Smoking Cessation Among Participants in the National Lung Screening Trial. JAMA Intern. Med..

[27] Caponnetto P., Campagna D., Ahluwalia J.S., Russell C., Maglia M., Riela P.M., Longo C.F., Busa B., Polosa R. (2023). Varenicline and Counseling for Vaping Cessation: A Double-Blind, Randomized, Parallel-Group, Placebo-Controlled Trial. BMC Med..

[28] Mungia R., Mexquitic M., Case K., Atique M., Jones B., MacCarthy D., Wang C.P. (2022). Implementation of a Youth and Young Adult E-Cigarette Cessation Program within a Dental Clinic Setting: A SToHN Feasibility Study. Tex. Dent. J..

[29] Kumbhalwar A., Hegde S., Kakodkar P., Mehta V., Gupte H., Jadhav S. (2022). Effectiveness of Behavioral Counseling in Smokeless Tobacco Cessation Among Adult Users Reporting to a Dental Hospital in Pune: A Randomized Controlled Trial. Cureus.

[30] Maria Minotti C. Walking Through Smoke: Implementation of the 5A’s for Smoking Cessation Counseling in Rural Virginia. https://digitalcommons.liberty.edu/cgi/viewcontent.cgi?article=3696&context=doctoral.

[31] Ferketich A.K., Pennell M., Seiber E.E., Wang L., Farietta T., Jin Y., Wewers M.E. (2014). Provider-Delivered Tobacco Dependence Treatment to Medicaid Smokers. Nicotine Tob. Res..

[32] Smith P.M., Sellick S.M., Spadoni M.M. (2012). Tobacco Cessation Clinical Practice Guideline Use by Rural and Urban Hospital Nurses: A Pre-Implementation Needs Assessment. BMC Nurs..

[33] Gomm M., Lincoln P., Egeland P., Rosenberg M. (2002). Helping hospitalised clients quit smoking: A study of rural nursing practice and barriers. Aust. J. Rural Health.

[34] Jafry M.Z., Martinez J., Chen T.A., Britton M., Martinez Leal I., Rogova A., Kyburz B., Williams T., Patel M., Carter B.J. (2023). Behavioral Health Care Provider’s Beliefs, Confidence, and Knowledge in Treating Cigarette Smoking in Relation to Their Use of the 5A’s Intervention. Addict. Behav. Rep..

[35] LaMorte W. The Health Belief Model. https://sphweb.bumc.bu.edu/otlt/mph-modules/sb/behavioralchangetheories/behavioralchangetheories2.html.

[36] LaMorte W. The Social Cognitive Theory. https://sphweb.bumc.bu.edu/otlt/mph-modules/sb/behavioralchangetheories/behavioralchangetheories5.html.

[37] Green D. Defining Rural Texas. https://comptroller.texas.gov/economy/fiscal-notes/archive/2023/aug/rural.php.

[38] United States Census Bureau 2010 Census Urban and Rural Classification and Urban Area Criteria. https://www.census.gov/programs-surveys/geography/guidance/geo-areas/urban-rural/2010-urban-rural.html.

[39] United States Census Bureau Urban and Rural. https://www.census.gov/programs-surveys/geography/guidance/geo-areas/urban-rural.html.

[40] Office of Management and Budget 2020 Standards for Delineating Core Based Statistical Areas. https://www.federalregister.gov/documents/2021/07/16/2021-15159/2020-standards-for-delineating-core-based-statistical-areas.

[41] Health Resource, Services Administration Defining Rural Population. https://www.hrsa.gov/rural-health/about-us/what-is-rural.

[42] Health Resource, Services Administration Rural Health Grants Eligibility Analyzer. https://www.usa.gov/agencies/health-resources-and-services-administration.

[43] United States Census Bureau Nation’s Urban and Rural Populations Shift Following 2020 Census. https://www.census.gov/newsroom/press-releases/2022/urban-rural-populations.html.

[44] Texas Department of Agriculture Texas County Designations. https://texasagriculture.gov/portals/0/forms/er/rural-metro%20counties.pdf.

[45] Texas Department of Housing and Community Affairs 2024 Index of Texas Counties. https://en.wikipedia.org/wiki/Texas_Department_of_Housing_and_Community_Affairs.

[46] NIHCM Foundation Rural Health: Addressing Barriers to Care. https://www.nihcm.org/publications/rural-health-addressing-barriers-to-care.

[47] Health Resource, Services Administration Glossary. https://www.google.com/search?q=Health+Resource+%26+Services+Administration.+Glossary.&sca_esv=0fabfdd21ee9e8db&ei=jFekZ5WJNZmb4-EP5cWK6QE&ved=0ahUKEwiV_6THtq6LAxWZzTgGHeWiIh0Q4dUDCBA&uact=5&oq=Health+Resource+%26+Services+Administration.+Glossary.&gs_lp=Egxnd3Mtd2l6LXNlcnAiNEhlYWx0aCBSZXNvdXJjZSAmIFNlcnZpY2VzIEFkbWluaXN0cmF0aW9uLiBHbG9zc2FyeS4yBBAAGEcyBBAAGEcyBBAAGEcyBBAAGEcyBBAAGEcyBBAAGEcyBBAAGEcyBBAAGEdI2ANQ4gJY4gJwAHgEkAEAmAEAoAEAqgEAuAEDyAEA-AEBmAIDoAIMmAMAiAYBkAYIkgcBM6AHAA&sclient=gws-wiz-serp.

[48] U.S. Department of Health, Human Services Health Professional Shortage Areas (HPSAs) and Medically Underserved Areas/Populations (MUA/P) Shortage Designation Types.

[49] Texas 2036. Rural Texas Is as Big as Our State’s Future. https://texas2036.org/future-of-rural/.

[50] U.S. Department of Health, Human Services Health Workforce Shortage Areas. https://data.hrsa.gov/topics/health-workforce/shortage-areas.

[51] Siddiqi A.D., Britton M., Chen T.A., Carter B.J., Wang C., Martinez Leal I., Rogova A., Kyburz B., Williams T., Patel M. (2022). Tobacco Screening Practices and Perceived Barriers to Offering Tobacco Cessation Services among Texas Health Care Centers Providing Behavioral Health Treatment. Int. J. Environ. Res. Public Health.

[52] Chan L., Hart L.G., Goodman D.C. (2006). Geographic Access to Health Care for Rural Medicare Beneficiaries. J. Rural Health.

[53] Mumford E.A., Stillman F.A., Tanenbaum E., Doogan N.J., Roberts M.E., Wewers M.E., Chelluri D. (2019). Regional Rural-Urban Differences in E-Cigarette Use and Reasons for Use in the United States. J. Rural Health.

[54] Samaha H.L., Correa-Fernández V., Lam C., Wilson W.T., Kyburz B., Stacey T., Williams T., Reitzel L.R. (2017). Addressing Tobacco Use Among Consumers and Staff at Behavioral Health Treatment Facilities Through Comprehensive Workplace Programming. Health Promot. Pract..

[55] Texas Association of Rural Health Clinics Home. http://www.tarhc.org/.

[56] Texas Association of Community Health Centers Home. https://www.tachc.org/.

[57] Texas Association of Addiction Professionals Home. https://www.taap.org/.

[58] Siddiqi A.D., Chen T.A., Britton M., Martinez Leal I., Carter B.J., Correa-Fernández V., Rogova A., Kyburz B., Williams T., Casey K. (2023). Changes in Substance Use Treatment Providers’ Delivery of the 5A’s for Non-Cigarette Tobacco Use in the Context of a Comprehensive Tobacco-Free Workplace Program Implementation. Int. J. Environ. Res. Public Health.

[59] Jafry M.Z., Reuven S.M., Britton M., Chen T.A., Martinez Leal I., Rogova A., Kyburz B., Williams T., Patel M., Reitzel L.R. (2022). Providers’ Non-Cigarette Tobacco Use Intervention Practices in Relation to Beliefs about Patients, Prioritization of and Skills for Intervention, and Referral Knowledge in Texas Healthcare Centers Providing Care to Persons with Behavioral Health Needs. Int. J. Environ. Res. Public Health.

[60] Le K., Chen T.A., Martinez Leal I., Correa-Fernández V., Obasi E.M., Kyburz B., Williams T., Casey K., Taing M., O’Connor D.P. (2021). Organizational Factors Moderating Changes in Tobacco Use Dependence Care Delivery Following a Comprehensive Tobacco-Free Workplace Intervention in Non-Profit Substance Use Treatment Centers. Int. J. Environ. Res. Public Health.

[61] Carter B.J., Siddiqi A.D., Chen T.A., Britton M., Martinez Leal I., Correa-Fernández V., Rogova A., Kyburz B., Williams T., Casey K. (2023). Educating Substance Use Treatment Center Providers on Tobacco Use Treatments Is Associated with Increased Provision of Counseling and Medication to Patients Who Use Tobacco. Int. J. Environ. Res. Public Health.

[62] Garey L., Neighbors C., Leal I.M., Lam C.Y., Wilson W.T., Kyburz B., Stacey T., Correa-Fernández V., Williams T., Zvolensky M.J. (2019). Tobacco-Related Knowledge Following a Comprehensive Tobacco-Free Workplace Program within Behavioral Health Facilities: Identifying Organizational Moderators. Patient Educ. Couns..

[63] Rural Health Information Hub Healthcare Access in Rural Communities. https://www.ruralhealthinfo.org/topics/healthcare-access.

[64] Rural Health Information Hub Recruitment and Retention for Rural Health Facilities. https://www.ruralhealthinfo.org/topics/rural-health-recruitment-retention.

[65] Knudsen J., Williams A., Perry S. Tennessee Workforce Survey 2004. https://www.google.com/search?q=Knudsen%2C+J.%3B+Williams%2C+A.%3B+Perry%2C+S.+Tennessee+Workforce+Survey+2004&sca_esv=0fabfdd21ee9e8db&ei=8FekZ8HDCMiF4-EPo82UoAw&ved=0ahUKEwjB-8_2tq6LAxXIwjgGHaMmBcQQ4dUDCBA&uact=5&oq=Knudsen%2C+J.%3B+Williams%2C+A.%3B+Perry%2C+S.+Tennessee+Workforce+Survey+2004&gs_lp=Egxnd3Mtd2l6LXNlcnAiREtudWRzZW4sIEouOyBXaWxsaWFtcywgQS47IFBlcnJ5LCBTLiBUZW5uZXNzZWUgV29ya2ZvcmNlIFN1cnZleSAyMDA0SLcQUABYuA9wAHgAkAEAmAEAoAEAqgEAuAEDyAEA-AEC-AEBmAIAoAIAmAMAkgcAoAcA&sclient=gws-wiz-serp.

[66] Texas Department of State Health Services Quitline Services. https://www.yesquit.org/services.htm.

[67] Britton M., Rogova A., Chen T.A., Martinez Leal I., Kyburz B., Williams T., Patel M., Reitzel L.R. (2023). Texas Tobacco Quitline Knowledge, Attitudes, and Practices within Healthcare Agencies Serving Individuals with Behavioral Health Needs: A Multimethod Study. Prev. Med. Rep..

[68] Nitturi V., Chen T.A., Martinez Leal I., Correa-Fernández V., Drenner K., Kyburz B., Williams T., Obasi E.M., Britton M., Howard M. (2021). Implementation and Outcomes of a Train-the-Trainer Program at Behavioral Health Treatment Centers as a Mechanism to Maintain Organizational Capacity to Address Tobacco Use Disorder. Int. J. Environ. Res. Public Health.

[69] Weinberger A.H., Gbedemah M., Wall M.M., Hasin D.S., Zvolensky M.J., Goodwin R.D. (2018). Cigarette Use Is Increasing among People with Illicit Substance Use Disorders in the United States, 2002–2014: Emerging Disparities in Vulnerable Populations. Addiction.

[70] Marynak K., VanFrank B., Tetlow S., Mahoney M., Phillips E., Jamal A., Schecter A., Tipperman D., Babb S. (2018). Tobacco Cessation Interventions and Smoke-Free Policies in Mental Health and Substance Abuse Treatment Facilities—United States, 2016. MMWR Morb. Mortal. Wkly. Rep..

[71] Rojewski A.M., Bailey S.R., Bernstein S.L., Cooperman N.A., Gritz E.R., Karam-Hage M.A., Piper M.E., Rigotti N.A., Warren G.W. (2019). Considering Systemic Barriers to Treating Tobacco Use in Clinical Settings in the United States. Nicotine Tob. Res..

[72] Ziedonis D.M., Guydish J., Williams J., Steinberg M., Foulds J. (2006). Barriers and Solutions to Addressing Tobacco Dependence in Addiction Treatment Programs. Alcohol Res. Health.

[73] Conrad M., Bolte T., Gaines L., Avery Z., Bodie L. (2018). The Untreated Addiction: Going Tobacco-Free in a VA Substance Abuse Residential Rehabilitation Treatment Program (SARRTP). J. Behav. Health Serv. Res..

[74] Taylor G.M., Lindson N., Farley A., Leinberger-Jabari A., Sawyer K., Te Water Naudé R., Theodoulou A., King N., Burke C., Aveyard P. (2021). Smoking Cessation for Improving Mental Health. Cochrane Database Syst. Rev..

[75] Stein R.J., Pyle S.A., Haddock C.K., Poston W.S.C., Bray R., Williams J. (2008). Reported Stress and Its Relationship to Tobacco Use among U.S. Military Personnel. Mil. Med..

[76] Lemon S.C., Friedmann P.D., Stein M.D. (2003). The Impact of Smoking Cessation on Drug Abuse Treatment Outcome. Addict. Behav..

[77] Van Amsterdam J., Van Den Brink W. (2022). Smoking As an Outcome Moderator In the Treatment of Alcohol Use Disorders. Alcohol Alcohol..

[78] Morris C.D., Garver-Apgar C.E. (2020). Nicotine and Opioids: A Call for Co-Treatment as the Standard of Care. J. Behav. Health Serv. Res..

[79] Edmond M.B., Aletraris L., Roman P.M. (2015). Rural Substance Use Treatment Centers in the United States: An Assessment of Treatment Quality by Location. Am. J. Drug Alcohol Abus..

[80] Pullen E., Oser C. (2014). Barriers to Substance Abuse Treatment in Rural and Urban Communities: Counselor Perspectives. Subst. Use Misuse.

[81] Knudsen H.K., Roman P.M. (2004). Modeling the Use of Innovations in Private Treatment Organizations: The Role of Absorptive Capacity. J. Subst. Abus. Treat..

[82] Cofta-Woerpel L., Lam C., Reitzel L.R., Wilson W., Karam-Hage M., Beneventi D., Cofer J., Baker E., Wetter D.W., Cinciripini P.M. (2018). A Tele-Mentoring Tobacco Cessation Case Consultation and Education Model for Healthcare Providers in Community Mental Health Centers. Cogent Med..

[83] Sharac J., Markus A., Tolbert J., Rosenbaum S. Community Health Centers in a Time of Change: Results from an Annual Survey. https://files.kff.org/attachment/Issue-Brief-Community-Health-Centers-in-a-Time-of-Change-Results-from-an-Annual-Survey.pdf.

[84] Odahowski C.L., Crouch E.L., Zahnd W.E., Probst J.C., McKinney S.H., Abshire D.A. (2021). Rural-Urban Differences in Educational Attainment among Registered Nurses: Implications for Achieving an 80% BSN Workforce. J. Prof. Nurs..

[85] Martinez Leal I., Taing M., Correa-Fernández V., Obasi E.M., Kyburz B., Le K., Koshy L., Chen T.A., Williams T., Casey K. (2021). Addressing Smoking Cessation among Women in Substance Use Treatment: A Qualitative Approach to Guiding Tailored Interventions. Int. J. Environ. Res. Public Health.

[86] Pepper J.K., McRee A.-L., Gilkey M.B. (2014). Healthcare Providers’ Beliefs and Attitudes About Electronic Cigarettes and Preventive Counseling for Adolescent Patients. J. Adolesc. Health.

[87] Holden R.R., Weiner I.B., Craighead W.E. (2010). Social Desirability. The Corsini Encyclopedia of Psychology.

[88] Althubaiti A. (2016). Information Bias in Health Research: Definition, Pitfalls, and Adjustment Methods. JMDH.

